# Underweight Young Women Without Later Weight Gain Are at High Risk for Osteopenia After Midlife: The KOBE Study

**DOI:** 10.2188/jea.JE20150267

**Published:** 2016-11-05

**Authors:** Yukako Tatsumi, Aya Higashiyama, Yoshimi Kubota, Daisuke Sugiyama, Yoko Nishida, Takumi Hirata, Aya Kadota, Kunihiro Nishimura, Hironori Imano, Naomi Miyamatsu, Yoshihiro Miyamoto, Tomonori Okamura

**Affiliations:** 1 Department of Preventive Medicine and Epidemiology Informatics, National Cerebral and Cardiovascular Center, Suita, Osaka, Japan; 1 国立循環器病研究センター; 2 Foundation for Biomedical Research and Innovation, Kobe, Japan; 2 先端医療振興財団; 3 Department of Environmental and Preventive Medicine, Hyogo College of Medicine, Nishinomiya, Hyogo, Japan; 3 兵庫医科大学環境予防医学; 4 Department of Preventive Medicine and Public Health, Keio University, Shinjuku, Tokyo, Japan; 4 慶應義塾大学衛生学公衆衛生学; 5 Center for Supercentenarian Medical Research, Keio University School of Medicine, Shinjuku, Tokyo, Japan; 5 慶應義塾大学医学部百寿総合研究センター; 6 Center for Epidemiologic Research in Asia, Shiga University of Medical Science, Otsu, Japan; 6 滋賀医科大学アジア疫学研究センター; 7 Department of Social and Environmental Medicine, Graduate School of Medicine, Osaka University, Suita, Osaka, Japan; 7 大阪大学公衆衛生学; 8 Department of Clinical Nursing, Shiga University of Medical Science, Otsu, Japan; 8 滋賀医科大学臨床看護学

**Keywords:** bone mineral density, osteopenia, body mass index, underweight, 骨密度, 骨量減少, body mass index, 痩せ

## Abstract

**Background:**

Although underweight young women are targets for interventions to prevent low bone mineral density (BMD), the relationship between change in body mass index (BMI) from youth to older age and BMD has not been widely investigated in community dwellers.

**Methods:**

In 749 healthy Japanese women aged 40–74 years, BMD was measured by quantitative ultrasound and anthropometric measurements, and BMI was calculated from body weight and height. The BMI of participants at age 20 years was estimated by self-reported body weight and their present height. They were classified into four groups according to the presence of underweight (BMI <18.5 kg/m^2^) at 20 and/or at present. Logistic regression models were used to estimate multivariate-adjusted odds ratios (ORs) of the presence of underweight at 20 and/or at present for osteopenia (BMD T score <−1 standard deviations) compared with participants with BMI ≥18.5 kg/m^2^ both at 20 and at present.

**Results:**

The participants who were underweight both at 20 and at present had a higher OR for osteopenia compared with those with BMI ≥18.5 kg/m^2^ at 20 and at present (OR 3.94; 95% confidence interval [CI], 1.97–7.89). Those underweight only at present also had significantly increased OR of developing osteopenia (OR 2.95; 95% CI, 1.67–5.24). The OR of those underweight only at 20 was 0.87 (95% CI, 0.51–1.48).

**Conclusions:**

Current underweight was associated with increased risk for osteopenia among Japanese women, especially in those who were underweight both at 20 and at present. To prevent low BMD in the future, maintaining appropriate body weight might be effective for young underweight women.

## Abstract (ja)

**背景：:**

若年痩せ女性は将来の骨粗鬆症予防のターゲット集団であると考えられるが、地域住民において若年期以降の体重変化と骨密度との関連は明らかにされていない。

**方法：:**

都市部住民コホート研究「神戸トライアル」のベースライン調査（2010-11年度）を受けた40-75歳の女性749名を対象とした。調査時のbody mass index（BMI）は身長と体重の測定値より算出し、20歳時のBMIは調査時に聴取した20歳時の体重と調査時の身長から推定して、BMI18.5kg/m^2^未満を痩せとした。骨密度は調査時に踵骨で超音波法により測定し、Tスコア-1SD未満を低骨密度とした。20歳時・調査時ともに痩せでなかった群を対照に、20歳時かつ/または調査時に痩せであった群の、調査時に低骨密度であるリスクを、ロジスティック回帰分析により多変量調整オッズ比を算出して推定した。

**結果：:**

多変量調整オッズ比は、対照群に比べ、20歳時・調査時ともに痩せであった群で3.94 (95%信頼区間：1.97－7.89)と最も高く、また調査時のみ痩せだった群でも2.95 (1.67－5.24)と有意に高かった。20歳時のみ痩せで調査時は痩せでなかった群のオッズ比は0.87 (0.51－1.48)であった。

**結論：:**

若年期も中高年期も痩せでなかった群に比べ、どちらも痩せていた群では、中高年期に低骨密度であるリスクは有意に上昇したが、若年期は痩せでも中高年期に痩せでない群では、有意なリスクの上昇は認められなかった。若年期に痩せていても、それ以降にBMIが改善すれば、中高年期の低骨密度を予防できる可能性が示された。

## INTRODUCTION

Osteoporosis is a major risk factor for fractures in the elderly. In 2013, fractures were the fourth leading cause of disabilities that subsequently required support for daily living in Japan.^1^ The estimated prevalence of osteoporosis diagnosed in the proximal femur among people aged 40 years or over in Japan is 12.4% in men and 26.5% in women, and prevalence is higher in women than in men in every generation.^2^

Previous studies have reported significant positive associations between bone mineral density (BMD) and body weight or body mass index (BMI) in cross-sectional studies.^3^^–^^7^ In cohort studies, Tanaka et al reported that being underweight was a risk factor for osteoporotic fractures,^8^ and Forsmo et al reported that an increase in body weight was associated with higher BMD after 11 years of follow-up in middle-aged women.^9^

The prevalence of underweight among young women is increasing in Japan.^10^^,^^11^ The percentages of women in their twenties with BMI less than 18.5 kg/m^2^ were 13.5% in 1979, 20.4% in 1998, and 21.5% in 2013.^10^^,^^11^ These young underweight Japanese women are considered to have lower BMD compared with those who are not underweight. However, future weight gain might increase BMD in middle or older age.

Development of strategies for preventing future osteoporotic fractures in young underweight women requires investigating the relationship between BMD in middle or older age and BMI both in young and middle-aged or older women. However, few studies have investigated these associations in a general female population. We investigated the relationships between present BMD and BMI both at age 20 years and in middle and older age among healthy females.

## METHODS

### Study participants

Data of the present study were based on the baseline survey of the Kobe Orthopedic and Biomedical Epidemiological (KOBE) study. The details of the KOBE study have been described elsewhere.^12^^–^^15^ The baseline survey was performed between July 2010 and December 2011. Study participants were healthy volunteers aged 40–74 years who were residents of Kobe City, a major urban area in Japan. From 1117 participants in the baseline survey (341 men and 776 women), we excluded all male participants as well as the 27 female participants with missing values, resulting in 749 women that were included in the study. Written informed consent was obtained from each participant. The study was approved by the Ethics Committee of the Institute of Biomedical Research and Innovation (Committee approval number: 11-12).

### Data collection

Participants completed a questionnaire that included demographic characteristics, body weight at 20 years old, medical history, menopause status, postmenopausal years, dietary habit, nutritional supplement intake, walking time per day (less than 30, 30–59, 60–119, and 120 or more min), smoking habit (current, ex-, or never), and drinking habit (current, ex-, or never). Height and weight were measured with subjects wearing light clothing, and BMI was calculated as weight (kg) divided by the square of height (m). The BMI data from the survey are described as “present BMI” in the present study. The BMI at 20 years old was estimated using self-reported weight at age 20 and height at the time of the survey. BMD was measured using the quantitative ultrasound method in the right calcaneum (AOS-100NW; ALOKA Ltd., Tokyo, Japan). Blood samples were obtained from all participants and were tested in the commissioned clinical laboratory center (SRL Inc., Tokyo, Japan). Thyroid-stimulating hormone (TSH) was measured using electrochemiluminescence immunoassay, and subclinical hyperthyroidism was defined as TSH <0.5 µIU/mL.^16^

### Definitions of the main outcome

BMD was expressed in standard deviation (SD) units relative to the BMDs of young women (T score). Osteopenia was defined according to World Health Organization criteria as a T score less than −1 SD.^17^^,^^18^ Although osteoporosis is defined as a T score less than −2.5 SD, osteoporosis was not defined in the present study because there were too few participants with a T score less than −2.5 SD (*n* = 3).

### Statistical analysis

Participants were categorized into four groups as follows: Group 1 (normal or overweight^19^ with BMI ≥18.5 kg/m^2^ at 20 years old and at present), Group 2 (normal or overweight at 20 years old and underweight^19^ with BMI <18.5 kg/m^2^ at present), Group 3 (underweight at 20 years old and normal or overweight at present), and Group 4 (both underweight at 20 years old and at present). Characteristics among the four groups were compared by analysis of variance or by Kruskal-Wallis test for continuous variables and by Chi-square tests for categorical variables. Logistic regression models were used to estimate odds ratios (ORs) and 95% confidence intervals (CIs) of the participants who were underweight at 20 years old for osteopenia compared with those who were normal or overweight at 20 years old. The same analyses were performed using the participants’ present BMI. In these analyses, model 1 was adjusted for age and model 2 was adjusted for age, postmenopausal years, taking calcium supplements, walking time per day, smoking habit, drinking habit, milk or yogurt intake, and hyperthyroidism. Postmenopausal years were defined as zero for menstruating women. For the analysis of BMI at 20 years old, model 3 was adjusted for model 2 variables and present BMI category. For the analysis of present BMI, model 3 was adjusted for model 2 variables and BMI category at 20 years old.

Logistic regression models were also used to estimate ORs of Groups 2–4 for osteopenia compared with Group 1. Variables for adjustment in model 1 and 2 were the same as those mentioned above. The estimated OR of Group 4 for osteopenia was compared with Group 3 after adjusting for the variables in model 2 (additional model). These logistic regression models were also performed after exclusion of participants with BMI ≥25.0 kg/m^2^.

All data were analyzed using Stata, version 13 (IBM Corp., Armonk, NY, USA). All reported *P*-values were two-tailed, and *P* < 0.05 was considered statistically significant.

## RESULTS

Among all participants, mean (SD) age was 58.0 (8.7) years. The percentages of participants in menopause, currently smoking, currently drinking, and taking calcium supplement were 77.7%, 2.0%, 37.0%, and 5.2%, respectively. Of all participants, 302 (40%) had osteopenia.

Table 1 shows the characteristics of the four groups classified by the presence of underweight at 20 years old and at present. Mean age of participants in group 4 was relatively low. However, there were no significant differences among the four groups for any variables.

**Table 1.** Characteristics of participants according to the presence of underweight at 20 years old and/or at present

	**At 20 years old**				*P* value
	Normal or Overweight		Underweight		

	**Present**				
	Normal or Overweight	Underweight	Normal or Overweight	Underweight	

	Group 1	Group 2	Group 3	Group 4	
*n*	521	74	103	51	
Age	58.4 (8.5)	57.9 (8.1)	57.2 (9.3)	54.7 (9.5)	0.116
BMI at 20 years old					
Median (25th–75th percentile)	20.5 (19.6–21.8)	19.7 (19.2–20.6)	17.7 (17.2–18.1)	18.0 (16.9–18.3)	
Range	18.5–29.1	18.6–23.6	15.5–18.5	15.1–18.5	
BMI at present					
Median (25th–75th percentile)	21.4 (20.1–23.2)	17.7 (17.2–18.2)	20.2 (19.4–21.3)	17.7 (16.8–18.2)	
Range	18.5–39.6	13.6–18.5	18.5–26.6	15.2–18.5	
Menopause, %	78.7	79.7	77.7	64.7	0.142
Hyperthyroidism, %	2.7	1.4	1	2	0.683
Calcium supplement, %	4.4	6.8	9.7	2	0.096
Milk or yogurt intake, ml/week	1050 (600–1700)	1365 (560–1960)	1190 (500–1750)	990 (500–1400)	0.630
Walking time per day, %					0.305
less than 30 minutes	5.6	5.4	9.7	3.9	
30–59 minutes	25.9	24.3	23.3	33.3	
60–119 minutes	36.9	43.2	27.2	31.4	
120 minutes or more	31.7	27	39.8	31.4	
Smoking, %					0.354
Current, ex, never	2.3, 6.7, 91.0	1.4, 12.2, 86.5	0.0, 9.7, 90.3	3.9, 7.8, 88.2	
Alcohol drinking, %					0.988
Current, ex, never	37.2, 2.7, 60.1	36.5, 2.7, 60.8	35.0, 1.9, 63.1	39.2, 3.9, 56.9	

BMI, body mass index.

Normal or overweight: BMI ≥18.5 kg/m^2^, Underweight: BMI <18.5 kg/m^2^.

Continuous variables are presented as mean (standard deviation) or median (25th–75th percentile). Categorical variables are presented as percentage.

The ORs of being underweight at 20 years old for osteopenia are shown in Table 2. None of the ORs in any adjusted models were significantly increased. In model 3, after additional adjustment for the present BMI category (underweight or not underweight), the OR was slightly attenuated compared with the other models. In Table 2, the ORs of being underweight at present for osteopenia are also shown. The ORs were significantly increased in all models, and the multivariable-adjusted OR of being underweight was 3.39 (95% CI, 2.10–5.46) in model 3. In the analysis of women with BMI <25.0 kg/m^2^ either at 20 years old or at present (Table 3), the results were similar after multivariate adjustment (OR of being underweight at 20 years old, 0.93; 95% CI, 0.59–1.47), and the OR of being underweight at present was 3.23 (95% CI, 1.99–5.23).

**Table 2.** Multivariate-adjusted odds ratios of underweight for osteopenia according to BMI at 20 years old and at present

	*n*	Case (%)	OR (95% CI)		

			Model 1	Model 2	Model 3
BMI category at 20 years old					
Underweight	154	61 (39.6)	1.24 (0.82–1.88)	1.26 (0.83–1.93)	0.99 (0.63–1.54)^a^
Normal or overweight	595	241 (40.5)	ref	ref	ref

BMI category at present					
Underweight	125	69 (55.2)	3.29 (2.07–5.20)	3.38 (2.12–5.38)	3.39 (2.10–5.46)^b^
Normal or overweight	624	233 (37.3)	ref	ref	ref

BMI, body mass index; CI, confidence interval; OR, odds ratio.

Normal or overweight: BMI ≥18.5 kg/m^2^, Underweight: BMI <18.5 kg/m^2^.

Osteopenia: T score less than −1 standard deviation.

Model 1: Adjusted for age.

Model 2: Adjusted for age, postmenopausal years, taking calcium supplement, walking time per day, smoking habit, drinking habit, milk or yogurt intake and hyperthyroidism.

Model 3^a^: Adjusted for model 2 variables and BMI category at present.

Model 3^b^: Adjusted for model 2 variables and BMI category at 20 years old.

**Table 3.** Multivariate-adjusted odds ratios of underweight for osteopenia according to BMI at 20 years old and at present among women with BMI less than 25.0 kg/m^2^

	*n*	Case (%)	OR (95% CI)		

			Model 1	Model 2	Model 3
BMI category at 20 years old					
Underweight	151	60 (39.7)	1.14 (0.75–1.75)	1.18 (0.76–1.82)	0.93 (0.59–1.47)^a^
Normal weight	532	224 (42.1)	ref	ref	ref

BMI category at present					
Underweight	125	69 (55.2)	3.08 (1.94–4.91)	3.18 (1.99–5.09)	3.23 (1.99–5.23)^b^
Normal weight	558	215 (38.5)	ref	ref	ref

BMI, body mass index; CI, confidence interval; OR, odds ratio.

Normal weight: BMI 18.5–24.9 kg/m^2^, Underweight: BMI <18.5 kg/m^2^.

Osteopenia: T score less than −1 standard deviation.

Model 1: Adjusted for age.

Model 2: Adjusted for age, postmenopausal years, taking calcium supplement, walking time per day, smoking habit, drinking habit, milk or yogurt intake and hyperthyroidism.

Model 3^a^: Adjusted for model 2 variables and BMI category at present.

Model 3^b^: Adjusted for model 2 variables and BMI category at 20 years old.

The ORs of Groups 2–4 for osteopenia compared with Group 1 are shown in Table 4. Approximately one-third of the participants who were underweight at age 20 were also underweight at present. Compared with Group 1, the ORs of osteopenia in Groups 2 and 4 (OR 2.95; 95% CI, 1.67–5.24 and OR 3.94; 95% CI, 1.97–7.89, respectively) were significantly increased after multivariable adjustment. Group 3 had a lower risk of osteopenia than Group 1, although not significantly so (OR 0.87; 95% CI, 0.51–1.48). In the analyses of Groups 3 and 4, the OR of osteopenia in Group 4 compared with Group 3 were significantly increased (multivariable adjusted OR 3.79; 95% CI, 1.57–9.15). In the analysis of women with BMI <25.0 kg/m^2^ at age 20 and at present, the multivariable-adjusted ORs in Groups 2, 3, and 4 were 2.73 (95% CI, 1.53–4.87), 0.80 (95% CI, 0.46–1.38), and 3.66 (95% CI, 1.82–7.39), respectively (Table 5). In Group 4, the OR compared with Group 3 was 3.83 (95% CI, 1.56–9.41).

**Table 4.** Multivariate-adjusted odds ratios of the presence of underweight at 20 years old and/or present for osteopenia

	**At 20 years old**			
	Normal or Overweight		Underweight	

	**Present**			
	Normal or Overweight	Underweight	Normal or Overweight	Underweight

	Group 1	Group 2	Group 3	Group 4
*n*	521	74	103	51
Osteopenia (%)	199 (38.2)	42 (56.8)	34 (33.0)	27 (52.9)
OR (95% CI)				
Model 1	ref	2.87 (1.63–5.05)	0.88 (0.52–1.46)	3.86 (1.93–7.73)
Model 2	ref	2.95 (1.67–5.24)	0.87 (0.51–1.48)	3.94 (1.97–7.89)

Additional model	—	—	ref	3.79 (1.57–9.15)

CI, confidence interval; OR, odds ratio.

Normal or overweight: BMI ≥18.5 kg/m^2^, Underweight: BMI <18.5 kg/m^2^.

Osteopenia: T score less than −1 standard deviation.

Model 1: Adjusted for age.

Model 2: Adjusted for age, postmenopausal years, taking calcium supplement, walking time per day, smoking habit, drinking habit, milk or yogurt intake and hyperthyroidism.

Additional model: Participants with normal or overweight at 20 years were excluded, and the logistic regression model was adjusted for model 2 variables.

**Table 5.** Multivariate-adjusted odds ratios of the presence of underweight participants at 20 years old and/or osteopenia among women with BMI less than 25.0 kg/m^2^

	**At 20 years old**			
	Normal weight		Underweight	

	**Present**			
	Normal weight	Underweight	Normal weight	Underweight

	Group 1	Group 2	Group 3	Group 4
*n*	458	74	100	51
Osteopenia (%)	182 (39.7)	42 (56.8)	33 (33.0)	27 (52.9)
OR (95% CI)				
Model 1	ref	2.64 (1.49–4.67)	0.79 (0.47–1.34)	3.57 (1.77–7.19)
Model 2	ref	2.73 (1.53–4.87)	0.80 (0.46–1.38)	3.66 (1.82–7.39)

Additional model	—	—	ref	3.83 (1.56–9.41)

CI, confidence interval; OR, odds ratio.

Normal weight: BMI 18.5–24.9 kg/m^2^, Underweight: BMI <18.5 kg/m^2^.

Osteopenia: T score less than −1 standard deviation.

Model 1: Adjusted for age.

Model 2: Adjusted for age, postmenopausal years, taking calcium supplement, walking time per day, smoking habit, drinking habit, milk or yogurt intake and hyperthyroidism.

Additional model: Participants with normal weight at 20 years were excluded, and the logistic regression model was adjusted for model 2 variables.

## DISCUSSION

The present study showed that the OR of osteopenia in women who were underweight at age 20 years and at present compared with women whose weight was normal or overweight at age 20 years and at present were significantly higher. Also, the OR among women who were underweight at 20 years but normal or overweight at present was slightly lower; however, this difference was not significant. In addition, the OR among women who were normal or overweight at 20 years but underweight at present was significantly higher. These results did not change after exclusion of participants with ≥BMI 25.0 kg/m^2^ either at age 20 years or at present.

It has been reported that postmenopausal women with BMI <18.5 kg/m^2^ have low BMD and that they are at high risk of future fractures.^8^^,^^20^ Similarly, young underweight women with anorexia nervosa have also been reported to have low BMD compared with healthy young women.^21^ Consequently, underweight women should have low BMD regardless of age. Although the BMD measurement sites and severity of being underweight were different in these studies, their results are consistent with the present observations that women who remained underweight had low BMD.

Although there are few studies investigating the effects of weight gain on BMD among healthy young underweight women, some studies have investigated the effects among young women with anorexia nervosa.^22^^,^^23^ These studies demonstrated that weight gain does not increase BMD in this population. Further, the study by Misra et al^23^ reported that adolescent girls who did not recover from anorexia nervosa had decreased BMD compared with healthy controls, but girls who recovered from anorexia nervosa did not.^23^ Therefore, weight gain among underweight young women might prevent a decrease in BMD. In addition, it has been reported that weight gain increases BMD among perimenopausal women with BMI <23.0 kg/m^2^.^24^ Therefore, weight gain might increase or stabilize BMD among both young and middle-aged women. Although it is not clear in the present study when the participants with BMI <18.5 kg/m^2^ at age 20 years and with BMI ≥18.5 kg/m^2^ at present gained their weight, it is possible that there was no increase in the OR of osteopenia among these women compared with those with BMI ≥18.5 kg/m^2^ both at 20 years old and at present because of weight gain. In addition, the multivariable-adjusted OR for underweight at 20 years old was attenuated after adjustment for present BMI category, but the OR of underweight at present was not attenuated after adjustment for BMI at 20 years old (Table 2). This result indicates that BMD may be more strongly associated with BMI category at present than that at the age 20 years.

In the present study, one-third of the 154 participants who were underweight at 20 years old remained underweight at present. Because the number of young underweight women is increasing in Japan,^10^^,^^11^ the number of women who remain underweight in later life may increase in the future. As a consequence, the number of women who are at high risk for osteopenia may increase. In a study investigating the desire for thinness among 631 female university students in Japan, it was reported that nearly half of the underweight and 89% of normal-weight students desired to be thin.^25^ In addition, the National Nutrition Survey in Japan reported that the mean desired BMI values were 18.7 kg/m^2^ among young women aged 15–19 years and 19.2 kg/m^2^ in women aged 20–24 years.^26^ Because weight gain might prevent osteopenia but the desired body weight is low among underweight women, it is important to educate Japanese young women about a desirable healthy weight.

The present study showed that the OR for osteopenia among women with a BMI ≥18.5 kg/m^2^ at age 20 years but <18.5 kg/m^2^ at present was also high. Weight loss has been reported to lead to bone loss in previous studies,^11^^,^^27^ which is consistent with the present results. Thus, maintaining appropriate body weight not only at a young age but also after midlife is important to prevent osteopenia. However, aerobic and weight-bearing exercises are also recommended to prevent osteopenia.^28^ Therefore, if women who are overweight after midlife need to lose weight, these exercises rather than dietary restriction might be effective for improving health status.

The present study has several limitations. First, BMD was not measured by dual-energy X-ray absorptiometry or computed X-ray densitometry.^18^ Although quantitative ultrasound is not an established method for making a definitive diagnosis of osteopenia, it is useful as an examination procedure in screening for osteopenia.^18^^,^^29^ Second, BMD was measured only at the calcaneum, while it is usually measured at the lumbar spine, hip, femoral neck, calcaneum, forearm, or whole body. Of these sites, measurement of BMD at the hip or femoral neck is important, because these fracture sites are the leading cause of deterioration of daily living activities. Because the correlation between the BMD of the calcaneum and femoral neck has been reported to be 0.6 among middle-aged and older Japanese women,^29^ BMD measured at the calcaneum could be an index of risk for severe fractures. Similar investigations to the present study should be performed using BMD data in other bone sites, because significant correlations between BMI and BMD at many bone sites other than the calcaneus have been reported.^5^^,^^8^^,^^29^^,^^30^ Third, because body weight at age 20 years was based on self-reported weight, there might have been recall bias. However, the influence of inaccurate recall data might be reduced because we used dichotomized BMI information. Nevertheless, some of the normal or overweight women at 20 years old might have been wrongly classified into the underweight group, because it has been reported that self-reported weights are lower than measured weights.^31^ Thus, ORs of osteopenia among women who were underweight at 20 years old might have been underestimated. In addition, height for estimating the BMI at age 20 years was based on the present data measured at the baseline survey of the KOBE study. Finally, the participants of the present study were not representative of the general Japanese population because the participants of the KOBE study were relatively healthy individuals who did not take medication for diabetes, hypertension, or dyslipidemia and did not have a past history of cardiovascular diseases. Also, the mean Z-score of BMD, which is expressed in SD relative to the BMD of age-matched women was 0.33; in other words, the BMDs of the participants were slightly higher compared with those of age-matched Japanese women. Thus, the participants were considered to be highly health-conscious volunteers. Therefore, the results of the present study should be applied to the general population with caution.

In conclusion, being underweight at present was associated with increased risk for osteopenia in women, especially who were also underweight at age 20. On the other hand, the risk of osteopenia in women with underweight only at 20 years old was not increased compared with women whose weight was normal or overweight at age 20 years and at present. Maintaining appropriate body weight might be effective in preventing future low BMD among young underweight women.

## ONLINE ONLY MATERIAL

Abstract in Japanese.

## References

[1] Ministry of Health, Labour and Welfare. National Livelihood Survey 2013 [cited 2015 September 10]. Available from: http://www.mhlw.go.jp/toukei/saikin/hw/k-tyosa/k-tyosa13/.

[2] Yoshimura N, Muraki S, Oka H, Mabuchi A, En-Yo Y, Yoshida M, Prevalence of knee osteoarthritis, lumbar spondylosis, and osteoporosis in Japanese men and women: the research on osteoarthritis/osteoporosis against disability study. J Bone Miner Metab. 2009;27:620–8. 10.1007/s00774-009-0080-8 19568689

[3] Nakaoka D, Sugimoto T, Kaji H, Kanzawa M, Yano S, Yamauchi M, Determinants of bone mineral density and spinal fracture risk in postmenopausal Japanese women. Osteoporos Int. 2001;12:548–54. 10.1007/s001980170075 11527051

[4] Yahata Y, Aoyagi K, Okano K, Yoshimi I, Kusano Y, Kobayashi M, Metacarpal bone mineral density, body mass index and lifestyle among postmenopausal Japanese women: relationship of body mass index, physical activity, calcium intake, alcohol and smoking to bone mineral density: the Hizen-Oshima study. Tohoku J Exp Med. 2002;196:123–9. 10.1620/tjem.196.123 12002268

[5] Zhu K, Hunter M, James A, Lim EM, Walsh JP Associations between body mass index, lean and fat body mass and bone mineral density in middle-aged Australians: The Busselton Healthy Ageing Study. Bone. 2015;74:146–52. 10.1016/j.bone.2015.01.015 25652209

[6] Asaoka D, Nagahara A, Shimada Y, Matsumoto K, Ueyama H, Matsumoto K, Risk factors for osteoporosis in Japan: is it associated with Helicobacter pylori? Ther Clin Risk Manag. 2015;11:381–91. 10.2147/TCRM.S80647 25834453 PMC4358368

[7] Wang MC, Bachrach LK, Van Loan M, Hudes M, Flegal KM, Crawford PB The relative contributions of lean tissue mass and fat mass to bone density in young women. Bone. 2005;37:474–81. 10.1016/j.bone.2005.04.038 16040285

[8] Tanaka S, Kuroda T, Saito M, Shiraki M Overweight/obesity and underweight are both risk factors for osteoporotic fractures at different sites in Japanese postmenopausal women. Osteoporos Int. 2013;24:69–76. 10.1007/s00198-012-2209-1 23229467

[9] Forsmo S, Aaen J, Schei B, Langhammer A What is the influence of weight change on forearm bone mineral density in peri- and postmenopausal women? The health study of Nord-Trondelag, Norway. Am J Epidemiol. 2006;164:890–7. 10.1093/aje/kwj268 16887894

[10] Ministry of Health, Labour and Welfare. National Nutrition Survey 2013 [cited 2015 September 10]. Available from: http://www.mhlw.go.jp/file/04-Houdouhappyou-10904750-Kenkoukyoku-Gantaisakukenkouzoushinka/0000068070.pdf.

[11] Ministry of Health, Labour and Welfare. National Nutrition Survey 1998 [cited 2015 September 10]. Available from: http://www0.nih.go.jp/eiken/chosa/kokumin_eiyou/doc_year/1998/1998_kek05.pdf.

[12] Higashiyama A, Wakabayashi I, Kubota Y, Adachi Y, Hayashibe A, Nishimura K, Does high-sensitivity C-reactive protein or low-density lipoprotein cholesterol show a stronger relationship with the cardio-ankle vascular index in healthy community dwellers?: the KOBE study. J Atheroscler Thromb. 2012;19:1027–34. 10.5551/jat.13599 22785137

[13] Sugiyama D, Higashiyama A, Wakabayashi I, Kubota Y, Adachi Y, Hayashibe A, The Relationship between Lectin-Like Oxidized Low-Density Lipoprotein Receptor-1 Ligands Containing Apolipoprotein B and the Cardio-Ankle Vascular Index in Healthy Community Inhabitants: The KOBE Study. J Atheroscler Thromb. 2015;22:499–508. 10.5551/jat.26450 25374294

[14] Kubota Y, Higashiyama A, Imano H, Sugiyama D, Kawamura K, Kadota A, Serum polyunsaturated fatty acid composition and serum high-sensitivity C-reactive protein levels in healthy Japanese residents: The Kobe study. J Nutr Health Aging. 2015;19:719–28. 10.1007/s12603-015-0497-9 26193854 PMC12878957

[15] Hirata T, Higashiyama A, Kubota Y, Nishimura K, Sugiyama D, Kadota A, HOMA-IR Values are Associated With Glycemic Control in Japanese Subjects Without Diabetes or Obesity: The KOBE Study. J Epidemiol. 2015;25:407–14. 10.2188/jea.JE20140172 26005064 PMC4444494

[16] Sonan M, Hiraoka K, Yamada E, Watanabe S, Kobayashi S Fundamental and clinical evaluation of TSH and thyroid hormone measurement by electrochemiluminescence immunoassay system “Modular Analytics <EE>”. J Med. 2001;46:749–71 (in Japanese).

[17] Kanis JA Assessment of fracture risk and its application to screening for postmenopausal osteoporosis: synopsis of a WHO report. WHO Study Group. Osteoporos Int. 1994;4:368–81. 10.1007/BF01622200 7696835

[18] Soen S, Fukunaga M, Sugimoto T, Sone T, Fujiwara S, Endo N, ; Japanese Society for Bone and Mineral Research and Japan Osteoporosis Society Joint Review Committee for the Revision of the Diagnostic Criteria for Primary Osteoporosis Diagnostic criteria for primary osteoporosis: year 2012 revision. J Bone Miner Metab. 2013;31(3):247–57. 10.1007/s00774-013-0447-8 23553500

[19] WHO Expert Consultation Appropriate body-mass index for Asian populations and its implications for policy and intervention strategies. Lancet. 2004;363:157–63. 10.1016/S0140-6736(03)15268-3 14726171

[20] Beck TJ, Petit MA, Wu G, LeBoff MS, Cauley JA, Chen Z Does obesity really make the femur stronger? BMD, geometry, and fracture incidence in the women’s health initiative-observational study. J Bone Miner Res. 2009;24:1369–79. 10.1359/jbmr.090307 19292617 PMC2718796

[21] Fazeli PK, Klibanski A Bone metabolism in anorexia nervosa. Curr Osteoporos Rep. 2014;12:82–9. 10.1007/s11914-013-0186-8 24419863 PMC3936314

[22] Milos G, Spindler A, Rüegsegger P, Hasler G, Schnyder U, Laib A, Does weight gain induce cortical and trabecular bone regain in anorexia nervosa? A two-year prospective study. Bone. 2007;41:869–74. 10.1016/j.bone.2007.07.017 17825636

[23] Misra M, Prabhakaran R, Miller KK, Goldstein MA, Mickley D, Clauss L, Weight gain and restoration of menses as predictors of bone mineral density change in adolescent girls with anorexia nervosa-1. J Clin Endocrinol Metab. 2008;93:1231–7. 10.1210/jc.2007-1434 18089702 PMC2291495

[24] Lee HR, Hong SS, Lee SY, Cho YH, Park HJ, Jung DW, The Impact of Body Weight Change on Bone Mineral Density of the Lumbar Spine in Perimenopausal Women: A Retrospective, One-year Follow-up Study. Korean J Fam Med. 2011;32:219–25. 10.4082/kjfm.2011.32.4.219 22745857 PMC3383136

[25] Mase T, Miyawaki C, Kouda K, Fujita Y, Ohara K, Nakamura H Relationship of a desire of thinness and eating behavior among Japanese underweight female students. Eat Weight Disord. 2013;18:125–32. 10.1007/s40519-013-0019-x 23760840

[26] Hayashi F, Takimoto H, Yoshita K, Yoshiike N Perceived body size and desire for thinness of young Japanese women: a population-based survey. Br J Nutr. 2006;96:1154–62. 10.1017/BJN20061921 17181892

[27] Zibellini J, Seimon RV, Lee CM, Gibson AA, Hsu MS, Shapses SA, Does Diet-Induced Weight Loss Lead to Bone Loss in Overweight or Obese Adults? A Systematic Review and Meta-Analysis of Clinical Trials. J Bone Miner Res. 2015;30:2168–78. 10.1002/jbmr.2564 26012544

[28] Orimo H, Nakamura T, Hosoi T, Iki M, Uenishi K, Endo N, Japanese 2011 guidelines for prevention and treatment of osteoporosis—executive summary. Arch Osteoporos. 2012;7:3–20. 10.1007/s11657-012-0109-9 23203733 PMC3517709

[29] Iida T, Chikamura C, Aoi S, Ikeda H, Matsuda Y, Oguri Y, A study on the validity of quantitative ultrasonic measurement used the bone mineral density values on dual-energy X-ray absorptiometry in young and in middle-aged or older women. Radiol Phys Technol. 2010;3:113–9. 10.1007/s12194-010-0086-x 20821084

[30] Kim SJ, Yang WG, Cho E, Park EC Relationship between Weight, Body Mass Index and Bone Mineral Density of Lumbar Spine in Women. J Bone Metab. 2012;19:95–102. 10.11005/jbm.2012.19.2.95 24524039 PMC3780918

[31] Yoong SL, Carey ML, D’Este C, Sanson-Fisher RW Agreement between self-reported and measured weight and height collected in general practice patients: a prospective study. BMC Med Res Methodol. 2013;13:38. 10.1186/1471-2288-13-38 23510189 PMC3599990

