# Supplementary material for: Underweight Young Women Without Later Weight Gain Are at High Risk for Osteopenia After Midlife: The KOBE Study
Source: J Epidemiol. 2016 Nov 5;26(11):572–8. doi: 10.2188/jea.JE20150267 (PMC5083320; doi:10.2188/jea.JE20150267)
Supplement: Abstract in Japanese. [file je-26-572-s001.pdf]

若年・中高年期ともに痩せの女性は、中高年期の低骨密度リスクが高い：神戸トライアル

辰巳友佳子[1,2]、東山綾[1,2]、久保田芳美[2,3]、杉山大典[2,4]、西田陽子[2]、平田匠[2,5]、門田文[2,6]、西村邦宏[1,2]、今野弘規[2,7]、宮松直美[2,8]、宮本恵宏[1,2]、岡村智教[2,4]

[1]国立循環器病研究センター
[2]先端医療振興財団
[3]兵庫医科大学環境予防医学
[4]慶應義塾大学衛生学公衆衛生学
[5]慶應義塾大学医学部百寿総合研究センター
[6]滋賀医科大学アジア疫学研究センター
[7]大阪大学公衆衛生学
[8]滋賀医科大学臨床看護学

背景：若年痩せ女性は将来の骨粗鬆症予防のターゲット集団であると考えられるが、地域住民において若年期以降の体重変化と骨密度との関連は明らかにされていない。
方法：都市部住民コホート研究「神戸トライアル」のベースライン調査（2010-11年度）を受けた40-75歳の女性749名を対象とした。調査時のbody mass index（BMI）は身長と体重の測定値より算出し、20歳時のBMIは調査時に聴取した20歳時の体重と調査時の身長から推定して、BMI18.5kg/m^2未満を痩せとした。骨密度は調査時に踵骨で超音波法により測定し、Tスコア-1SD未満を低骨密度とした。20歳時・調査時ともに痩せでなかった群を対照に、20歳時かつ/または調査時に痩せであった群の、調査時に低骨密度であるリスクを、ロジスティック回帰分析により多変量調整オッズ比を算出して推定した。
結果：多変量調整オッズ比は、対照群に比べ、20歳時・調査時ともに痩せであった群で3.94(95%信頼区間：1.97－7.89)と最も高く、また調査時のみ痩せだった群でも2.95（1.67－5.24)と有意に高かった。20歳時のみ痩せで調査時は痩せでなかった群のオッズ比は0.87（0.51－1.48)であった。
結論：若年期も中高年期も痩せでなかった群に比べ、どちらも痩せていた群では、中高年期に低骨密度であるリスクは有意に上昇したが、若年期は痩せでも中高年期に痩せでない群では、有意なリスクの上昇は認められなかった。若年期に痩せていても、それ以降にBMIが改善すれば、中高年期の低骨密度を予防できる可能性が示された。

キーワード：骨密度、骨量減少、body mass index、痩せ
